# Shall We Sweat the Crippled Soldier?

**Published:** 1916-05-27

**Authors:** 


### SHALL WE SWEAT THE CRIPPLED SOLDIER?

If a proper scale of pensions for the permanently disabled soldier is to be placed beyond the reach of injustice disguised as economy, that scale must be fought for, and secured, while public opinion is hot. The proper pension for the permanently disabled will be gained now, or it never will be gained at all. Even when it is gained, it must be secured against reductions or abatements. They will come, be sure, in a very insidious guise. Here is one side of this question which needs watching. The number of seriously disabled men is becoming so large that great efforts are being made to relieve their condition. Oral surgery, massage, medical gymnastics, the so-called medical "sculpture," are all at work. But we must not forget that it is costing money; and that the money so spent is not regarded, whoever is responsible for finding it, as coming under the sacred headings unquestionably provided for by the great spending departments.

Therefore the efforts now made to equip disabled men for some sort of human activity, preferably of a productive kind, must be watched very anxiously in the interest of the cripples. Such specialised skill as they may acquire under medical tuition must never be used as an excuse for reducing their pensions merely because they can make a little money by it. The notion, too, already heard, that the disabled should be ashamed of their pensions, and grasp at every chance to give them up, is a monstrous perversion of justice. The disabled should stick to their pensions as fast as they formerly stuck to their guns, and their countrymen should be equally proud of them for doing it.

If the disabled men are entitled to everything which medical aid can do for them, they are entitled to such skill as it can give them; and if they are entitled to the skill, they are certainly entitled to such money as they can make by these ingenious but freak activities. It must never be forgotten that though a disabled musician, say, may be taught to play the violin with his toes, he is none the less permanently disabled. His new skill is a necessity to be deplored—not a faculty to be exploited. Any profits ultimately accruing to him from this source must not be held as reducing the obligations of the Pensions Board towards the sufferer. To "take them into consideration," in the odious official phrase, in granting the pension would be an enormity, a crime, of which only a committee could be capable. It has not actually been committed yet, so far as we know, but some remarks lately dropped by a colonel of the R.A.M.C. are terribly sufficient to show which way the official wind is blowing. Let the public once more, therefore, spring to the defence of the disabled and rescue them from what, if it occurred, would be a disgraceful exploitation of such skilled makeshift employments as the medical profession may have been able to supply. It rests with public opinion alone, in the long run, to ensure that the permanently disabled shall not be robbed of part of their pensions by a process which, whatever official name is used to cloak it, would be nothing but sweating the disabled soldier.

